# The GR-gp78 Pathway is involved in Hepatic Lipid Accumulation Induced by Overexpression of 11β-HSD1

**DOI:** 10.7150/ijbs.42376

**Published:** 2022-04-30

**Authors:** Mengliang Hu, Tingting Han, Qiyu Pan, Dongsheng Ni, Fengyi Gao, Liying Wang, Hangjiang Ren, Xiaoyi Zhang, Haoyun Jiao, Yuefeng Wang, Dapeng Dai, Yong Man, Weiqing Tang, Yue Sun, Wei Li, Jian Li, Guoping Li

**Affiliations:** 1 The Key Laboratory of Geriatrics, Beijing Institute of Geriatrics, Institute of Geriatric Medicine, Chinese Academy of Medical Sciences, Beijing Hospital/National Center of Gerontology of National Health Commission, Beijing 100730, P.R. China; 2 Institute of Reproductive Health and Perinatology, Guangzhou Women and Children's Medical Center, Guangzhou Medical University, Guangzhou 510623, P.R. China; 3 State Key Laboratory of Stem Cell and Reproductive Biology, Institute of Zoology, Chinese Academy of Sciences, Beijing 100101, P.R. China; 4 Philips Institute for Oral Health Research, School of Dentistry and Massey Cancer Center, Virginia Commonwealth University, Richmond, Virginia 23298, USA; 5 College of Biology and Food, Shangqiu Normal University, Shangqiu, Henan 476000, P.R. China; 6 Graduate School of Peking Union Medical College, Beijing 100730, P.R. China; 7 Peking University Fifth School of Clinical Medicine (Beijing Hospital), Beijing 100730, P.R. China

**Keywords:** 11β-HSD1, glucocorticoid receptor, gp78, Insig2/SREBP1, non-alcoholic fatty liver disease.

## Abstract

Glucocorticoids are essential participants in the regulation of lipid metabolism. On a tissue-specific level, glucocorticoid signal is controlled by 11β-Hydroxysteroid dehydrogenase 1 (11β-HSD1). Up-regulation of 11β-HSD1 expression during non-alcoholic fatty liver disease (NAFLD) has been previously shown, while 11β-HSD1 inhibition has been shown to reduce hepatic lipids in NAFLD, but the underlying mechanisms remain unclear. Here, in this study, we created *in vitro* cell culture and *in vivo* transgenic hepatocyte-specific 11β-HSD1 mouse models of NAFLD to determine the regulatory mechanisms of 11β-HSD1 during lipid metabolism dysfunction. We found that 11β-HSD1 overexpression activated glucocorticoid receptors and promoted their nuclear translocation, and then stimulating gp78. The induction of gp78 sharply reduced expression of Insig2, but not Insig1, which led to up-regulation of lipogenesis regulatory proteins including SREBP1, FAS, SCD1, and ACC1. Our results suggested that overexpression of 11β-HSD1 induced lipid accumulation, at least partially through the GR/gp78/Insig2/SREBP1 pathway, which may serve as a potential diagnostic and therapeutic target for treatment of NAFLD.

## Introduction

Metabolic (dysfunction)-associated fatty liver disease (MAFLD), previously known as non-alcoholic fatty liver disease (NAFLD), influences about a quarter of the world's adult population, brings a major health and economic burden to all societies^1^-^5^. In order to be consistent with the cited literature and facilitate reading, the term NAFLD is still used in this paper. The diagnosis of NAFLD is defined by a triglyceride content exceeding 5% of the weight of the liver^2^. A clear understanding of the genetic factors underlying the changes in lipid metabolism that accompanies NAFLD is necessary to effectively manage this disease in clinical practice, and may further help to resolve its origins and to develop predictive markers of disease outcome^6^, ^7^.

Recent studies have identified an array of cellular processes and signalling pathways that are disrupted or otherwise adversely affected by NAFLD-associated dysfunction in lipid metabolism^8^, ^9^. An increasing body of evidence suggests that the dysregulation of 11β-Hydroxysteroid dehydrogenase 1 (11β-HSD1) was related to other conditions such as obesity, type 2 diabetes, and metabolic syndrome. For example, diet-induced obesity and associated metabolic dysfunction were shown to be mitigated in 11β-HSD1 knockout mice^10^. Furthermore, studies conducted by Seckl *et.al* have highlighted the potential for 11β-HSD1 inhibition as a therapeutic treatment in adipose and liver tissues^11^. Although genetic and dietary models for fatty liver disease have revealed a critical relationship between hepatic steatosis and 11β-HSD1 function, the mechanistic details of this association remain poorly understood.

Indeed, recently published results have shown that inhibition of hepatic 11β-HSD1 mediates a reduction in lipogenesis via the sterol regulatory element-binding protein (SREBP)-1 transcription factor and fatty acid synthase (FAS) that results in protection from steatosis and dyslipidemia in mice^12^. Many findings prove that SREBP-1c is a master regulator of lipogenesis and that an increase in *de novo* lipogenesis can lead to retention of excess triglycerides 13.

The activation of lipogenic gene expression is mediated by SREBP following degradation of its inhibitors Insulin Induced Gene 1/2 (Insig1 and Insig2)^14^-^16^. Glycoprotein 78 (gp78), a rate-limiting E3 ubiquitin ligase regulator of lipid metabolism, plays a critical role in the degradation of Insig1/2 to de-repress SRBEP^17^, ^18^. Thus, gp78 activity has detrimental effects on lipid biosynthesis. By decreasing the levels of lipid biosynthesis, the ablation of gp78 potentially benefits patients with metabolic diseases^19^. Lipid homeostasis is also modulated by an intricate process that requires translocation of glucocorticoid receptor (GR) into the nucleus where it acts as a transcriptional regulator of lipid biosynthesis pathway-associated genes in response to glucocorticoid (GC) binding^20^. It is worth noting that 11β-HSD1 has been shown to locally activate GCs, which may be a strong, underlying contributor to changes in lipid metabolism^21^.

Here, in this study, we developed cell and transgenic mouse models for NAFLD to investigate the potential pathophysiological roles and regulatory mechanisms of 11β-HSD1, in association with GR, gp78, and Insig1/2. Through *in vivo* and *in vitro* studies of protein expression and triglyceride accumulation, we observed the effects of 11β-HSD1 overexpression on the accumulation of lipids in liver tissue and hepatocytes. This work provides evidences for the critical role of 11β-HSD1 in lipid metabolism dysfunction, and can serve as a basis for the development of therapeutic targets for clinical treatment of NAFLD.

## Materials and Methods

### Materials

The anti-GR antibody (24050-1-AP), anti-gp78 antibody (16675-1-AP), anti-Insig2 antibody (24766-1-AP), anti-GAPDH antibody (60004-1-Ig), anti-carnitine palmitoyl transferase 1α (CPT1α) antibody (15184-1-AP), horseradish peroxidase (HRP) -conjugated Affinipure Goat Anti-Mouse IgG(H+L) (SA00001-1), HRP-conjugated Affinipure Goat Anti-Rabbit IgG(H+L) (SA00001-2) and HRP-conjugated Affinipure Rabbit Anti-Goat IgG(H+L) (SA00001-4) were purchased from Proteintech (Wuhan, China). The anti-FAS antibody (3180S), anti-Stearoyl-CoA desaturase-1 (SCD1) antibody (2438S), anti- Acetyl-CoA carboxylase 1 (ACC1) antibody (4190S), anti-Adipose triglyceride lipase (ATGL) antibody (2439S), anti-CD36 antibody (14347S) and anti-Phospho-Glucocorticoid Receptor (Ser211) antibody (4161S) were purchased from Cell Signaling Technology (Danvers, MA, USA). The anti-SREBP1 antibody (sc-13551), anti-peroxisome proliferator activated receptor α (PPARα) antibody (sc-398394), anti-Histone H3 antibody (sc-517576) and Ubiquitin antibody (sc-8017) were purchased from Santa Cruz Biotechnology (CA, USA). The anti-microsomal triglyceride transfer protein (MTTP) antibody (612022) was purchased from BD Biosciences (CA, USA). The anti-low density lipoprotein receptor (LDLR) antibody (3839) was purchased from Biovision (Milpitas, CA, USA). The anti-Insig1 antibody (ab70784) was were purchased from Abcam (Cambridge, UK). The anti-11β-HSD1 antibody (AF3397) was purchased from R&D System (Abingdon, UK). The anti-β-actin antibody (TA-09), anti-β-tubulin antibody (TA-10) were purchased from Beijing Zhongshan Jinqiao Biotechnology (Beijing, China). The BCA protein concentration test kit (23225) was purchased from Thermo Fisher Scientific (Waltham, MA, USA). Oleic acid (OA) (O1008), palmitic acid (PA) (P5585) and bovine serum albumin (BSA) (A1933) were purchased from Sigma-Aldrich (Missouri, USA). BVT2733 (HY-18054) was purchased from MCE (MedChem Express, New Jersey, USA). Cycloheximide (CHX) (S7418) and MG132 (S2619) were purchased from Selleckchem (Houston, TX, USA). 1.1 × T3 Super PCR Mix (TSE030) was purchased from Tsingke Biotechnology Co., Ltd (Beijing, China). Collagenase (17104019) was purchased from Gibco (Carlsbad, CA, USA). Rat tail collagen (C8062) was purchased from Beijing Solarbio Science & Technology Co., Ltd (Beijing, China).

### Generation of transgenic mice with hepatocyte-specific 11β-HSD1 expression

Transgenic mice with hepatocyte-specific expression of human 11β-HSD1 (pRP.EX2d-ALB-11β-HSD1) driven by the albumin promoter were generated by Cyagen Biosciences Co. (Guangzhou, China). The C57BL/6J genetic background was used to create the transgenic founder mice by cloning human 11β-HSD1 cDNA into the pRP.Des2d vector. The linearized pRP.EX2d-ALB-11β-HSD1 was separated from uncut plasmids by gel electrophoresis and purified using a QIAquick Gel extraction kit (Qiagen, Chatsworth, CA, USA), then adjusted to a final concentration of 3 ng/μl in Tris-EDTA buffer. To prepare for transformation, female C57BL/6J mice were hormonally super-ovulated and mated with male C57BL/6J mice. The following morning, the fertilized single cell eggs were collected from the oviducts and microinjected with the DNA solution under a microscope. The injected, fertilized eggs were transplanted into the oviducts of pseudo-pregnant C57BL/6J mice. The transgene-positive founder mice were bred with C57BL/6J mice to generate the transgenic mice used for experiments. Genotyping was performed by polymerase chain reaction with the following primers for 11β-HSD1 (323-bp product): forward 5'- CAG ATG GCA AAC ATA CGC AAG -3' and reverse 5'- TCT GGT CTG AAT TCC TCG TT -3'. Hepatocyte-specific 11β-HSD1 transgenic male mice (TGM) were used for all experiments and their non-transgenic male littermates were used as controls (Con).

Animals were housed in 20-22 °C, with 45-55% humidity and fed standard chow (Beijing HFK Bioscience Co., Ltd., Beijing, China) ad libitum. Liver tissues from each experimental group of male mice were collected at 5-6 months of age. All animal experiments were performed in accordance with recommendations in the National Research Council Guide for Care and Use of Laboratory Animals, with the protocols approved by Animal Care and Use Committee of Beijing Hospital, the Ministry of Health of China.

### Adenoviral transduction of Hep1-6 cells

The recombinant adenovirus containing 11β-HSD1 (Ad-11β-HSD1) was constructed by Cyagen Biosciences Co. (Guangzhou, China) and then amplified and purified in 293A cells that were cultured in basic DMEM (Gibco, Waltham, MA, USA) containing 10% fetal bovine serum. A recombinant adenovirus containing green fluorescent protein (Ad-GFP) was used as a control.

### Cell culture and treatment

Hep1-6 cells were cultured in high-glucose Dulbecco's modified Eagle medium (DMEM) (Invitrogen, Carlsbad, California, USA) containing 10% fetal bovine serum (Gibco, Grand Island, New York, USA), 100 units/ml penicillin (Invitrogen, Carlsbad, California, USA) and 0.1 mg/ml streptomycin (HyClone, Logan, Utah, USA), incubated at 37°C, 5% humidity, and 5% carbon dioxide concentration. The cells were collected for experiments during the exponential growth phase. Cells (2 × 10^5^) were seeded into 6-well plates and cultured 12 h, then a mixture 300 μM oleic acid/palmitic acid (OA/PA, 2∶1, M/M) was added and the cells were incubated for an additional 12 h, following a previously reported modified method^22^. At 2 h prior to the addition of adenovirus, 0.25 μM BVT2733 (dissolved in DMSO) was added to inhibit the activity of 11β-HSD1 and the inhibitor was present in the medium during the following time. An equal volume of DMSO was added to the medium as a BVT2733-treated control group. Then, the Ad-11β-HSD1 and Ad-GFP constructs were transfected separately into the cells and incubated for 36 h before harvesting protein samples. Each experiment was carried out for at least 3 times separately.

### Hepatocyte isolation and cultivation

Cultured primary mouse hepatocytes were isolated from the livers of 5-6 months TGM and Con, as described elsewhere^23^. Briefly, mice were euthanized, and the liver was removed and perfused with buffer A (1 × HBSS, no calcium, no magnesium, no phenol red with 0.5 mM EDTA, pH 7.4). Hepatocytes were released by perfusion in buffer B (buffer A without EDTA, supplemented with 5 mM CaCl_2_ and 0.3 mg/ml collagenase) with gentle palpation of the liver capsule during perfusion. And perfusion with buffer B stopped when the liver became slightly translucent and the surface no longer sprang back. Hepatocytes were washed once in cold DMEM medium with 10% FBS. Viable hepatocytes were counted using trypan blue and plated in rat tail collagen-coated six-well dishes to be cultured.

### Western blot analysis

To measure protein expression by Western blot analysis, extracts of liver tissues (20 μg of proteins) and cells (15 μg of proteins) were electrophoretically separated on SDS-PAGE gels, then subsequently transferred to a PVDF membrane (Millipore, Boston, Massachusetts, USA), blocked with 5% skim milk, and probed with primary antibodies at 4 °C overnight. Following three washes with Tris-Buffered Saline Tween-20, the membrane was incubated at room temperature with an HRP-conjugated anti-rabbit or anti-mouse secondary antibody for 2 h, followed by imaging with an ECL Plus detection system (Millipore, Boston, Massachusetts, USA) according to the manufacturer's instructions.

### Oil Red O staining

To observe lipid accumulation in tissues and cells by Oil Red O staining, cells were treated for 12 h with a mixture of 300 μM OA/PA (2:1, M/M) and then Ad-GFP or Ad-11β-HSD1 was added for another 36 h incubation. Cells were washed three times with 1 × PBS (5 min per wash), and fixed with 4% paraformaldehyde at room temperature for 15 min. Cells were dehydrated for 15 s with 60% isopropanol, then stained with prewarmed Oil Red O for 30 min at 37 °C before a final wash in 60% isopropanol. Haematoxylin was used to stain the nucleus. Coverslips were mounted with glycerol gelatin and observed under a microscope (Nikon Eclipse 80, imaging software NIS-Elements F Version 4.60.00). For tissue samples, frozen sections (7 μm) of liver specimens were placed on a glass slide for Oil Red Staining. After fixed in 4% paraformaldehyde at room temperature for 10 min, sections were briefly rinsed softly with 1 × PBS with then with 60% isopropanol, and then stained with Oil Red O for 20 min at room temperature. The next steps were as the same as those described above.

### Triglyceride measurement

To determine the triglyceride content in liver tissues and cells, lipids were extracted using a modified Folch and Lees extraction method^24^. Mice were fasted 5 ~ 6 h before sacrificed. Briefly, the 50-60 mg liver tissues or 2 × 10^6^ cells were added in glass tube with 6 ml of chloroform/methanol (v/v = 2∶1) solution overnight for lipids extraction. After adding 1.2 ml of diluted 0.05% H_2_SO_4_, the mixture was centrifuged, and 1 ml of chloroform phase was then dried down with 1 ml of chloroform containing 1% Triton X-100 under nitrogen. The lipid extract was dissolved in deionized water after drying, and the triglyceride content was measured using a colorimetric lipid quantification enzyme kit (Prodia Diagnostics Co., Ltd, Germany).

### Immunofluorescence staining

Cells were placed on glass cover slips for 24 h and then fixed with 4% paraformaldehyde and permeabilized using 0.1% Triton X-100. An anti-rabbit primary antibody (GR or p-GR, 1∶1000) was applied. An Alexa Fluor 594-Conjugated Goat anti-Rabbit IgG(H+L) or a Alexa Fluor 488-Conjugated Goat anti-Rabbit IgG (H+L) was used (1:200). Hoechst 33258 was used to identify the cell nuclei. Immunofluorescence microscopy was conducted with a Nikon A1 Plus microscope (Nikon, Minato, Tokyo, Japan) to observe the activation of GR/p-GR. NIS-Elements imaging software version 4.40 was used to image acquisition. Researchers were blinded to this process.

### Nuclear and cytosolic separation

Separation of the nuclear and cytosolic fractions was performed using the Nuclear and Cytoplasmic Protein Extraction Kit (Sangon Biotech, Shanghai, China) according to the manufacturer's instructions.

### siRNA Transfection

Negative control siRNA (NC siRNA, sense: 5'-UUC UCC GAA CGU GUC ACG UTT-3', antisense: 5'-ACG UGA CAC GUU CGG AGA ATT-3') and gp78 siRNA (sense: 5'- GGA CUU CAG UGA GGU AGA ATT -3', antisense: 5'- UUC UAC CUC ACU GAA GUC CTT -3') were designed and synthesized from Genepharma (Shanghai, China). Lipofectamine™ 2000 Reagent was purchased from Invitrogen and transfection complexes were prepared according to the manufacturers protocol. After primary mouse hepatocytes were incubated in 300 μM OA/PA for 12 h, the culture supernatants were changed with *Opti*-MEM (Gibco Inc., Carlsbad, CA, United States) and the transfection complex was added directly to the culture dish. After 8 h, the medium was replaced with fresh culture medium. Primary mouse hepatocytes were harvested and collected 48h after siRNA transfection. Then we measure protein expression by western blot analysis.

### Chase experiments

Cycloheximide (CHX) was purchased from Selleckchem (Houston, TX, USA) and dissolved in DMSO. After cells were incubated in 300 μM OA/PA for 12 h, the primary hepatocytes switched to the same fresh medium containing CHX (100 µM final concentration). At the indicated time after CHX treatment, cells were harvested and protein expression was measured by western blot analysis.

### Ubiquitination assay by immunoprecipitation

Primary mouse hepatocytes were cultured as described previously. Endogenous protein ubiquitination assays were performed according to Xie *et.al*^25^. MG132 (10 µM final concentration) was added to the medium 12 h before harvesting the cells. Primary mouse hepatocytes were lysed with 1% SDS buffer (50 mM Tris-HCl pH 7.5, 150 mM NaCl, 1% SDS) and denatured by heating for 30 min. The lysates were diluted with lysis buffer (50 mM Tris-HCl pH 7.5, 150 mM NaCl, 1 mM EDTA, 1% NP-40, 1 mM PMSF and protease inhibitor cocktail (Roche)), so that the concentration of SDS was decreased to 0.1%. The diluted lysates were immunoprecipitated with an anti-Insig2 antibody (Proteintech, Wuhan, China, 24766-1-AP) or a Rabbit IgG antibody (Proteintech, Wuhan, China, B900610) overnight at 4 °C, which was followed by adding protein A/G agarose (Santa Cruz Biotechnology, CA, USA, sc-2003) to the lysates for 2 h at 4 °C. Immunocomplexes were washed with lysis buffer three times and then subjected to immunoblotting analyses.

### Statistics

Data were expressed as means ± SEM. Student's t-test was used to compare the means of two independent groups. One-way ANOVA was used to analyse differences in the means of more than two groups. A P value less than 0.05 was considered significant.

## Results

### Overexpression of 11β-HSD1 leads to hepatic lipid accumulation in Hep1-6 cells

**Figure 1:**
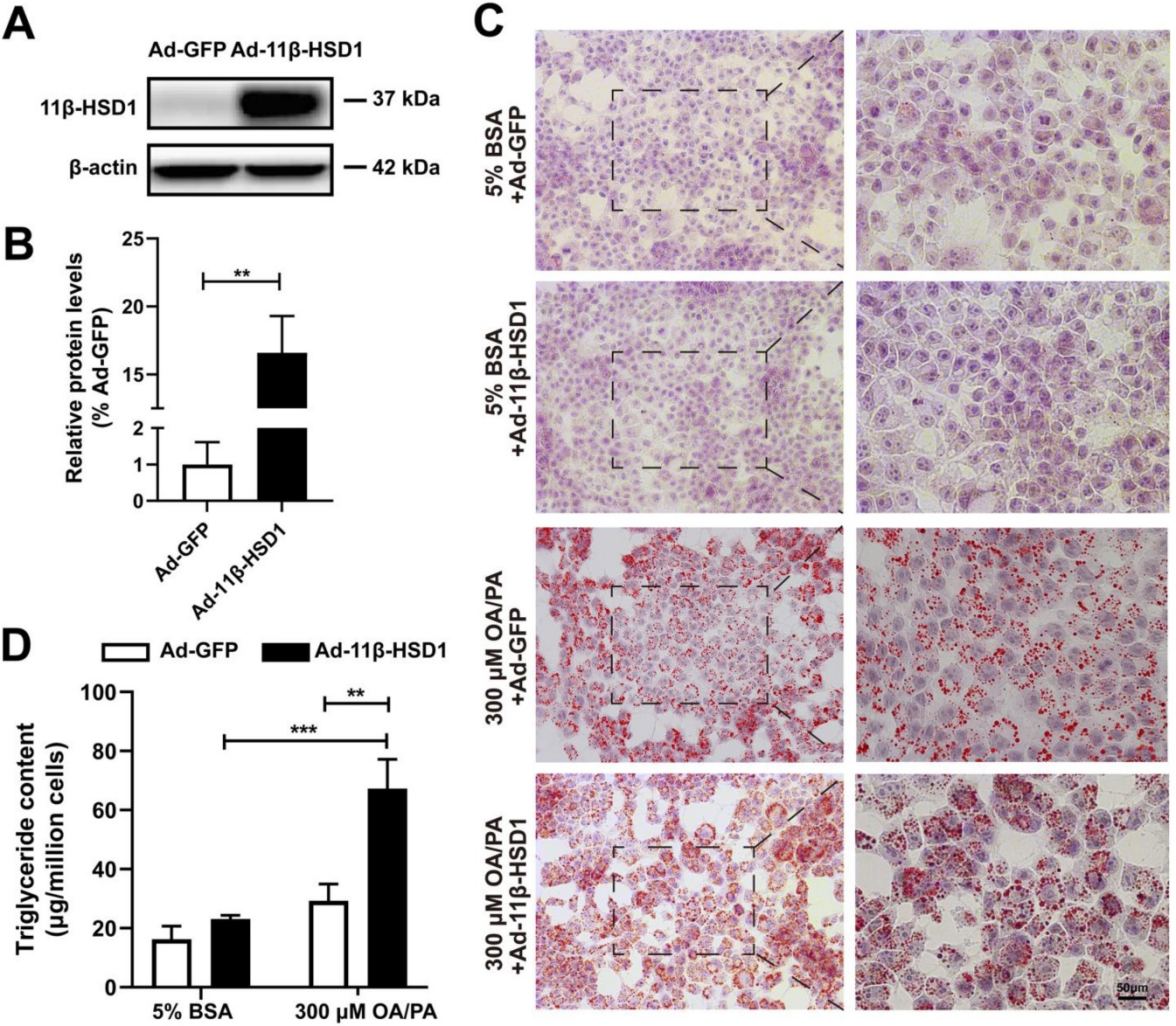
** Lipid and triglyceride content increased in Hep1-6 cells that overexpressed 11β-HSD1.** (A) Western blot showing the expression of 11β-HSD1 and (B) relative protein levels (n = 5). (C) Oil red O staining of Hep1-6 cells with 5% BSA or 300 μM OA/PA treatment. Box indicates magnified field of view. (D) Measurement of triglyceride content in Hep1-6 cells with the same treatment as (C) (n = 3). **P<0.01, ***P<0.001.

In order to explore the role of 11β-HSD1 expression in lipid metabolism, we first transfected Hep1-6 cells with Ad-11β-HSD1 and observed the effects of its overexpression on lipid accumulation. At 36 h post transfection, the cells successfully overexpressed 11β-HSD1 (1.00 ± 0.61 vs 16.59 ± 2.70, n = 5, *P*<0.01) compared with control group, which was transfected with Ad-GFP (Figure 1A-B). Oil red O staining of cells from the control group confirmed that the cells contained small lipid droplets with 300 μM OA/PA treatment, whereas in striking contrast, substantial lipid accumulation was observed in the Ad-11β-HSD1 treatment group (Figure 1C). Consistent with above results, in the presence of OA/PA, intracellular triglyceride levels increased (Figure 1D).

### Overexpression of 11β-HSD1 upregulates gp78 expression, stimulates Insig2 degradation and affects lipogenesis, which can be prevented by BVT2733

**Figure 2:**
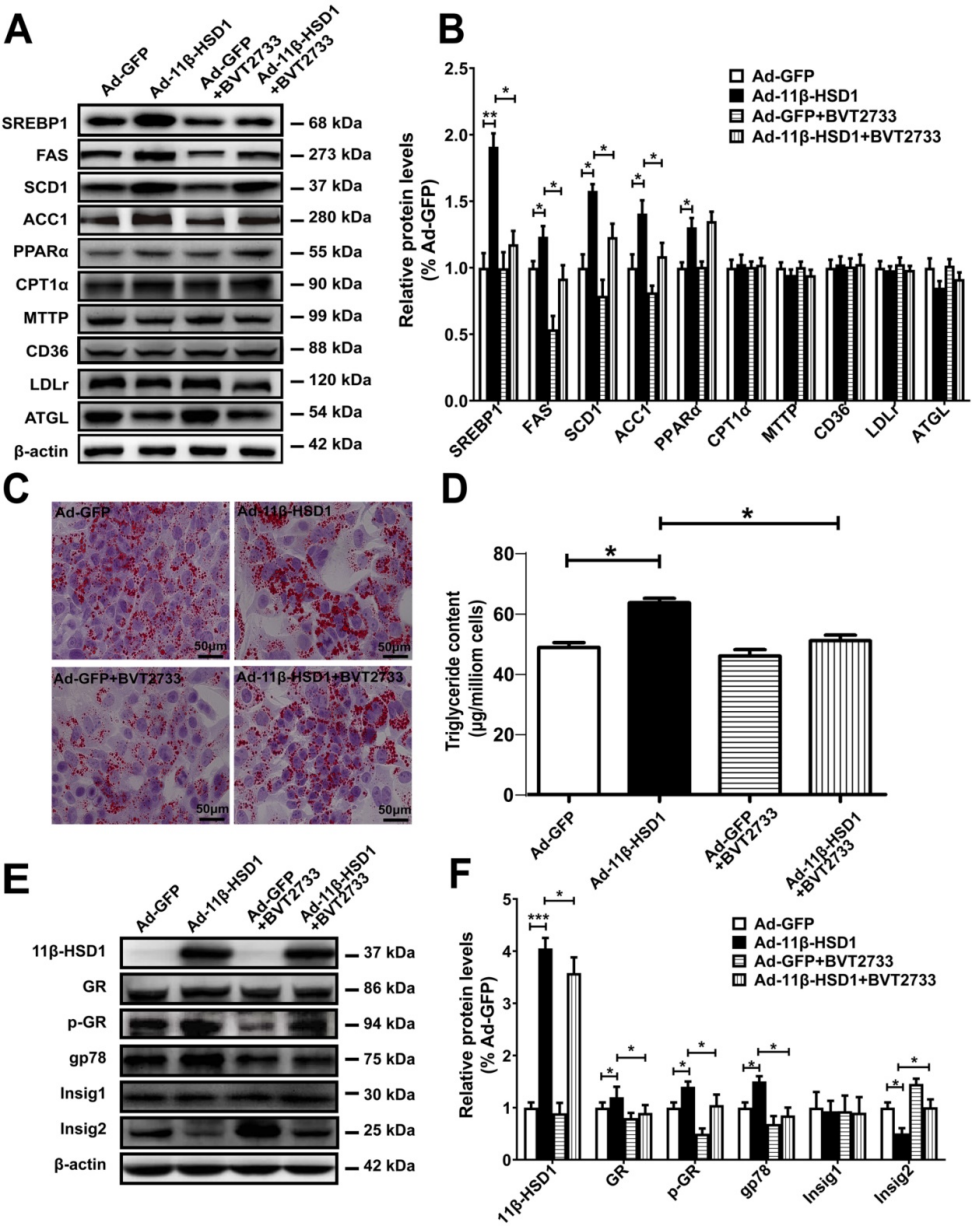
** Lipogenesis pathway is activated by overexpression of 11β-HSD1.** (A) Western blot detection of proteins related to the lipid biosynthesis, oxidation, secretion, uptake, and hydrolysis pathways in 11β-HSD1 overexpressed or GFP overexpressed Hep1-6 cells exposed or not to 0.25 μM BVT2733 and (B) relative protein levels (n = 3). (C) Oil red O staining of Hep1-6 cells and treatments from (A). (D) Measurement of triglyceride levels in Hep1-6 cells and treatments from (A) (n = 3). (E) Western blot showing the expression levels of lipogenesis pathway regulatory proteins in Hep1-6 cells and treatments from (A), and (F) relative protein levels (n = 3). *P<0.05, **P<0.01, ***P<0.001.

To investigate whether the activation of 11β-HSD1 affects lipid metabolism *in vitro*, we examined expression of SREBP1, the primary regulatory protein governing the long-chain fatty acid synthesis pathway, using Western blot analysis in 11β-HSD1-overexpressed Hep1-6 cells. Compared with the control group, mature SREBP1 peptide levels significantly increased in cells overexpressing 11β-HSD1 (Figure 2A-B). The downstream regulatory proteins FAS and SCD1 were also up-regulated (Figure 2A-B). Additionally, the expression of ACC1, which mediates the conversion from acetyl-CoA to malonyl-CoA, and thus plays a key role in the regulation of lipogenesis^26^, ^27^, was clearly increased (Figure 2A-B). We also examined protein expression in other pathways related to lipid metabolism, including lipid oxidation (PPARα and CPT1α)^28^, ^29^, secretion (MTTP)^30^, absorption (CD36 and LDLR)^30^, ^31^, and hydrolysis (ATGL)^32^, to determine if 11β-HSD1 interacts with other pathways to induce lipid accumulation in addition to affecting its biosynthesis. However, no observable difference was found in the expression of these components from other lipid-handling pathways, apart from a slight increase in PPARα (Figure 2A-B). Together, the results above demonstrate that intracellular lipid accumulation induced by 11β-HSD1 overexpression is primarily caused by activity of the lipid synthesis pathway, rather than by oxidation, secretion, absorption, or hydrolysis.

In addition, we treated cells with BVT2733, an inhibitor of 11β-HSD1, for 2 h prior to transfection with Ad-11β-HSD1 in Hep1-6 cells to determine if inhibition of 11β-HSD1 also suppresses the lipid biosynthetic pathway. Compared to Ad-11β-HSD1 expression group in the absence of the inhibitor, BVT2733 pre-treatment led to 0.7-, 0.5-, 0.3- and 0.4- fold decreases in SREBP1, FAS, SCD1, and ACC1 expression, respectively (Figure 2A-B). However, exposure to BVT2733 did not have a significant inhibitory effect on the expression of proteins related to lipid oxidation, secretion, absorption, or hydrolysis pathways (Figure 2A-B). Oil red O staining showed that Ad-11β-HSD1 overexpression induced lipid accumulation, and that this lipid deposition was largely reversed by BVT2733 treatment (Figure 2C). Consistent with the results, intracellular triglyceride content was restored to levels comparable to those in the Ad-GFP treatment group (Figure 2D). Collectively, these results indicate that BVT2733 inhibits 11β-HSD1 and attenuates lipid accumulation in Hep1-6 cells by suppressing SREBP1-stimulated lipogenesis.

To clarify the possible mechanisms by which 11β-HSD1 induced lipogenesis, using Hep1-6 cells we next examined the protein expression of GR, E3 ubiquitin-protein ligase gp78 (also known as AMFR) and Insig1/2, as well as the phosphorylation levels of GR, all of which are upstream of SREBP1. In agreement with the results presented above, overexpression of 11β-HSD1 significantly up-regulated the expression of GR, p-GR and gp78. More importantly, this enhancement of gp78 expression was associated with a significant reduction in Insig2, which was reduced by 50%, suggesting there was a subsequent release of Insig2 repression of SREBP1 (Figure 2E-F). However, the expression of Insig1 was unaffected. As expected, BVT2733 treatment abrogated 11β-HSD1-induced up-regulation of GR, p-GR, and gp78, in addition to 11β-HSD1-induced down-regulation of Insig2, but not Insig1 (Figure 2E-F).

### TGM accumulate lipid in liver tissues by activation of lipogenesis pathway

**Figure 3:**
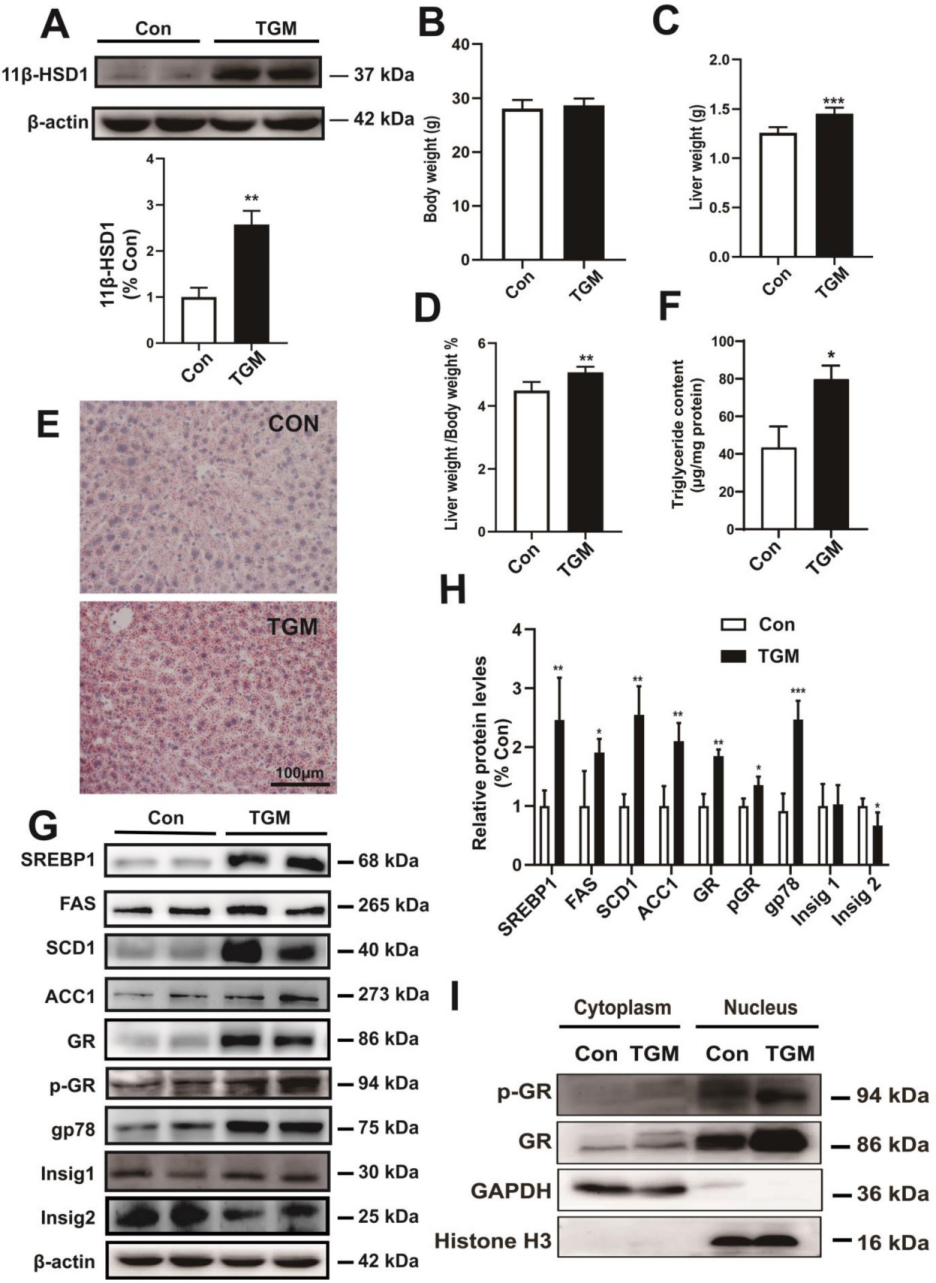
** The phenotype of hepatocyte-specific 11β-HSD1 transgenic mice (TGM).** (A) Western blot showing the expression of 11β-HSD1 in liver of Con vs TGM (n = 3). (B) Body weights (n = 10). (C) Liver weights (n = 10). (D) The statistical analysis of liver weight/body weight ratios (n = 10). (E) Oil red O staining of liver tissues. (F) Measurement of triglyceride levels in livers of Con vs TGM (n = 8). (G) Western blot detection of proteins related to lipogenesis and (H) relative protein levels (n = 3). (I) Western blot showing the expression levels of the GR and p-GR proteins in the nucleus and cytoplasm. *P<0.05, **P<0.01, ***P<0.001.

To better understand the precise regulatory mechanism of 11β-HSD1 *in vivo*, we created a hepatocyte-specific transgenic mouse (TGM) which overexpressed 11β-HSD1 specifically in liver tissues, untransformed male littermates were used as the control group (Con) (Figure 3A). We found no significant difference in body weight between the two groups (28.06 ± 1.61 g vs 28.69 ± 1.26 g, Con vs TGM, respectively; n = 10, Figure 3B). However, the liver weights were significantly higher in transgenic mice than in the control mice (1.26 ± 0.06 g vs 1.45 ± 0.06 g, Con vs TGM, n = 10, *P*<0.001, Figure 3C), and the percentage of liver weight/body weight was also higher (4.50% ± 0.28% vs 5.07% ± 0.18%, n = 10, *P*<0.01, Figure 3D). In addition, Oil red O staining assays (Figure 3E) showing enrichment of lipid droplets in liver tissues compared to Con, which was consistent with triglyceride content revealed a significant increase in liver tissues of TGM (43.41 ± 11.26 μg/mg protein vs 79.79 ± 7.26 μg/mg protein, n = 8, *P*<0.05, Figure 3F), further confirming hepatic lipid accumulation in hepatocyte-specific 11β-HSD1 transgenic mice.

Further detection of protein expression in liver tissues revealed that long-chain fatty acid synthesis-related regulatory proteins, including SREBP1, FAS, SCD1 and ACC1, were significantly up-regulated in hepatocyte-specific 11β-HSD1-overexpressing transgenic mice (Figure 3G-H). In addition, the levels of GR, p-GR, and gp78 increased concurrently with the down-regulation of Insig2, but not Insig1, suggesting that 11β-HSD1 affected the gp78/Insig2/SREBP1 pathway by activating GR, and finally led to hepatic lipid accumulation (Figure 3G-H). For GR is translocated to the nucleus to act as a transcription factor^33^, nuclear and cytoplasm separation of liver tissues was performed to determine changes in the local accumulation of GR and p-GR. Compared with Con, the expression of GR increased sharply in the nuclei of TGM liver tissue (Figure 3I). The level of p-GR in the nucleus was also elevated in TGM, demonstrating that 11β-HSD1 induced p-GR expression and translocation of GR to the nucleus (Figure 3I). Overall, both *in vivo* and *in vitro* assays of protein level reveal that 11β-HSD1 aggravates liver lipid accumulation.

### More lipids accumulated in primary hepatocytes derived from TGM

**Figure 4:**
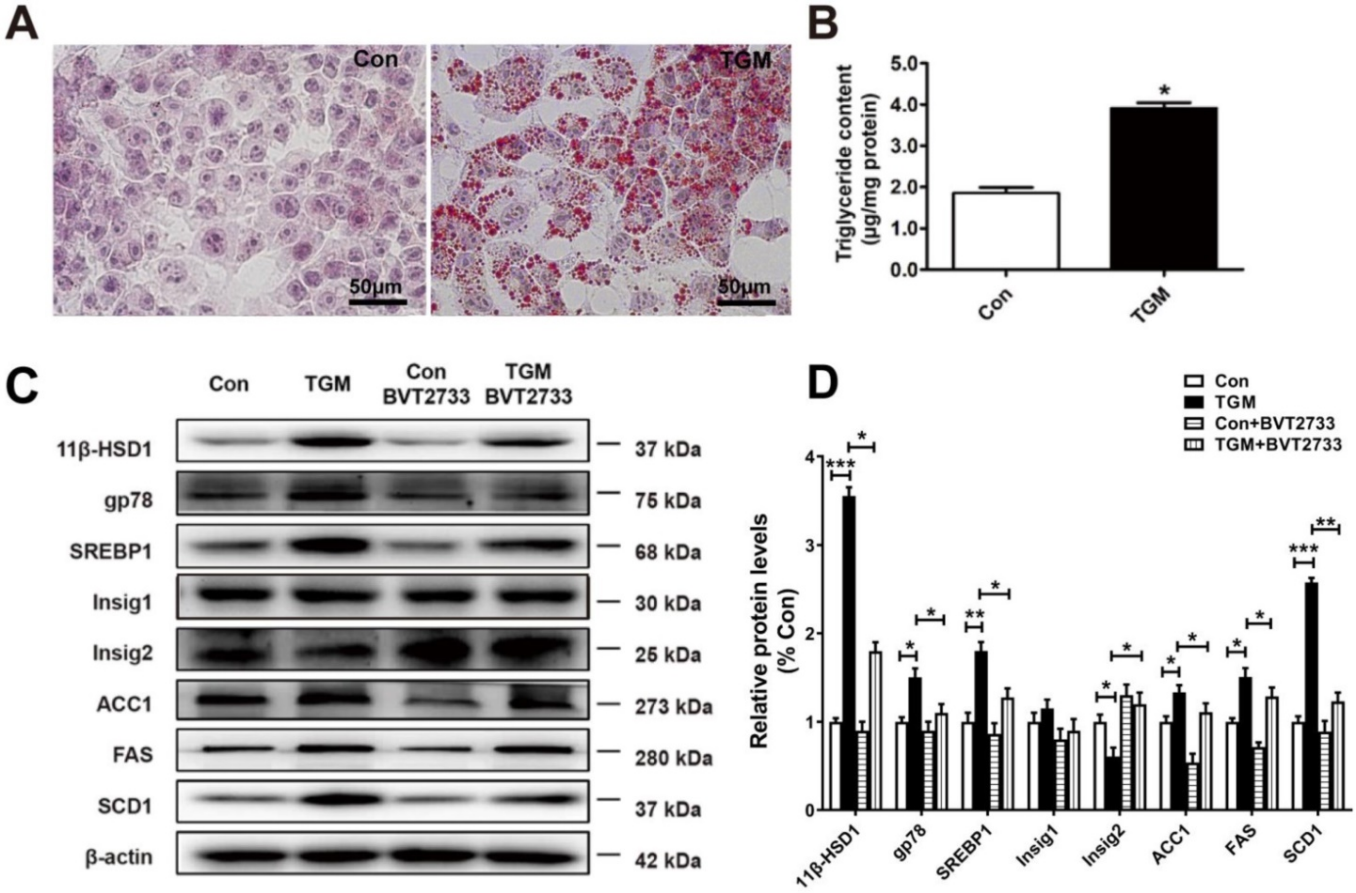
** Lipogenesis pathway is activated in primary hepatocytes derived from TGM.** (A) Oil red O staining of primary hepatocytes of Con vs TGM. Scale bars: 50 μm. (B) Measurement of triglyceride content in primary hepatocytes from Con vs TGM (n = 3). (C) Western blot showing the expression levels of lipogenesis pathway regulatory proteins in primary hepatocytes of Con vs TGM, treated or not with 0.25 μM BVT2733 and (D) relative protein levels (n = 3). *P<0.05, **P<0.01, ***P<0.001.

Similarly, we examined primary hepatocytes sampled from Con and TGM. Larger and more dense lipid droplets were observed by Oil red O staining in TGM primary hepatocytes (Figure 4A), while TG content in TGM primary hepatocytes was higher (1.85 ± 0.11 µg/mg protein vs 3.95 ± 0.20 µg/mg protein, n = 3, *P*<0.05, Figure 4B). Western blot detection of lipid biosynthesis pathway regulatory proteins in primary hepatocytes derived from TGM also showed that the expression of 11β-HSD1, gp78, SREBP1, FAS, ACC1, SCD1, and Insig2 were all significantly increased compared to the control group (Figure 4C-D). Furthermore, a significant increase in gp78, accompanied with reduced Insig2 in TGM untreated with BVT2733. After the addition of this inhibitor, the phenotype caused by 11β-HSD1 was partially reversed (Figure 4C-D). These experiments clarify that 11β-HSD1 regulates lipid synthesis through gp78/Insig2/SREBP1 pathway.

### Regulation of Insig2 by gp78 is a key component of lipid accumulation caused by overexpression of 11β-HSD1

**Figure 5:**
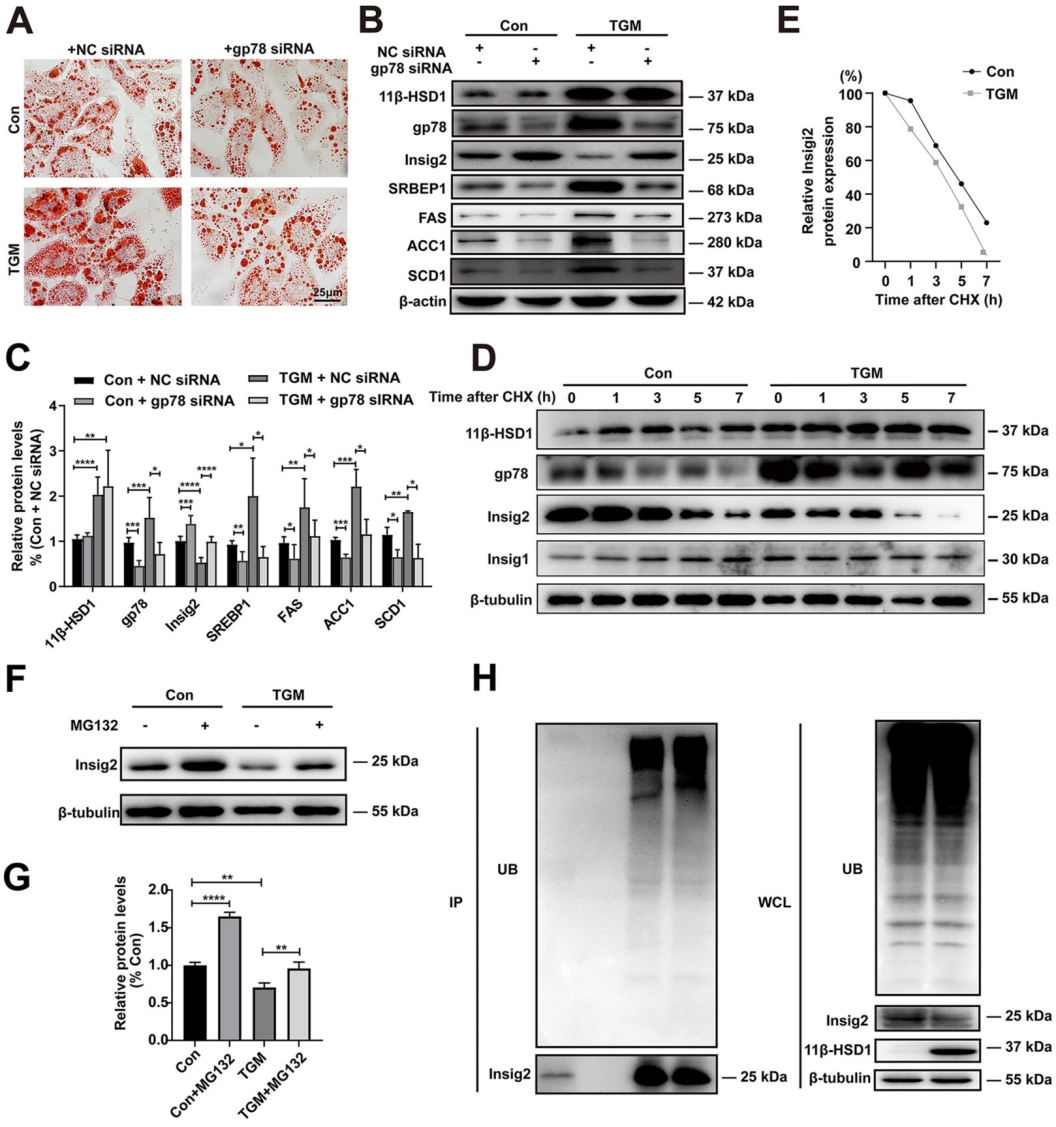
** The regulation of Insig2 by gp78 is vital to lipogenesis in primary hepatocytes derived from TGM.** (A) Oil red O staining of primary hepatocytes. (B) Western blot showing the expression levels of lipogenesis pathway regulatory proteins in primary hepatocytes of Con vs TGM after knocking down gp78 and (C) relative protein levels (n = 3). (D) Chase experiment showing that gp78 regulated Insig2 by affecting Insig2 stability. (E) Relative Insig2 protein expression of (D). (F) Western blot showing the expression levels of Insig2 in primary hepatocytes of Con vs TGM after treated or not with MG132 (10 μM final concentration) for 12h before harvest and (G) relative protein levels (n = 6). (H) Immunoprecipitation (IP) and western blotting analyses were performed to verify that Insig2 were degraded in ubiquitin pathway. (UB: Ubiquitin, WCL: Whole cell lysate.) *P<0.05, **P<0.01, ***P<0.001, ****P<0.0001.

In order to verify whether gp78-Insig2 signal axis plays a critical role in lipid accumulation caused by 11β-HSD1 overexpression, gp78 was knocked down by siRNA in the primary hepatocytes from Con or TGM. Consistent with results above, more and larger lipid droplets were observed in TGM than Con by Oil Red O Staining (Figure 5A). Compared with NC siRNA group, down-regulation of gp78 by specific siRNA rescued lipid accumulation (Figure 5A) and Insig2, SREBP1, FAS, SCD1 and ACC1 expression (Figure 5B-C) in hepatocytes from TGM. In general, the results of protein expression were consistent with the results of inhibiting 11β-HSD1 (Figure 5B and Figure 4C).

It has been proved that Insig2 is ubiquitylated by gp78^17^, ^19^. To verify this regulatory mechanism of Insig2 by gp78 in TGM, a series of experiments were applied. With the present of 100 µM CHX, the rate of Insig2 degradation is higher in TGM than in Con, indicating that gp78 regulated Insig2 by affecting the protein stability of Insig2 through chase experiment (Figure 5D-E). Reduced Insig2 in TGM could be rescued with the addition of the proteasome inhibitor MG132 (Figure 5F-G). And immunoprecipitation further confirmed Insig2 was degraded via ubiquitination, and more polyubiquitylated Insig2 were found in primary hepatocytes derived from TGM (Figure 5H).

Taken together, these results firmly verified that 11β-HSD1 induced lipogenesis by activating gp78, which led to the degradation of Insig2, and ultimately stimulated lipogenesis by accelerating the maturation of SREBP1.

### 11β-HSD1 promoted nuclear translocation of GR and following hepatic lipid accumulation

**Figure 6:**
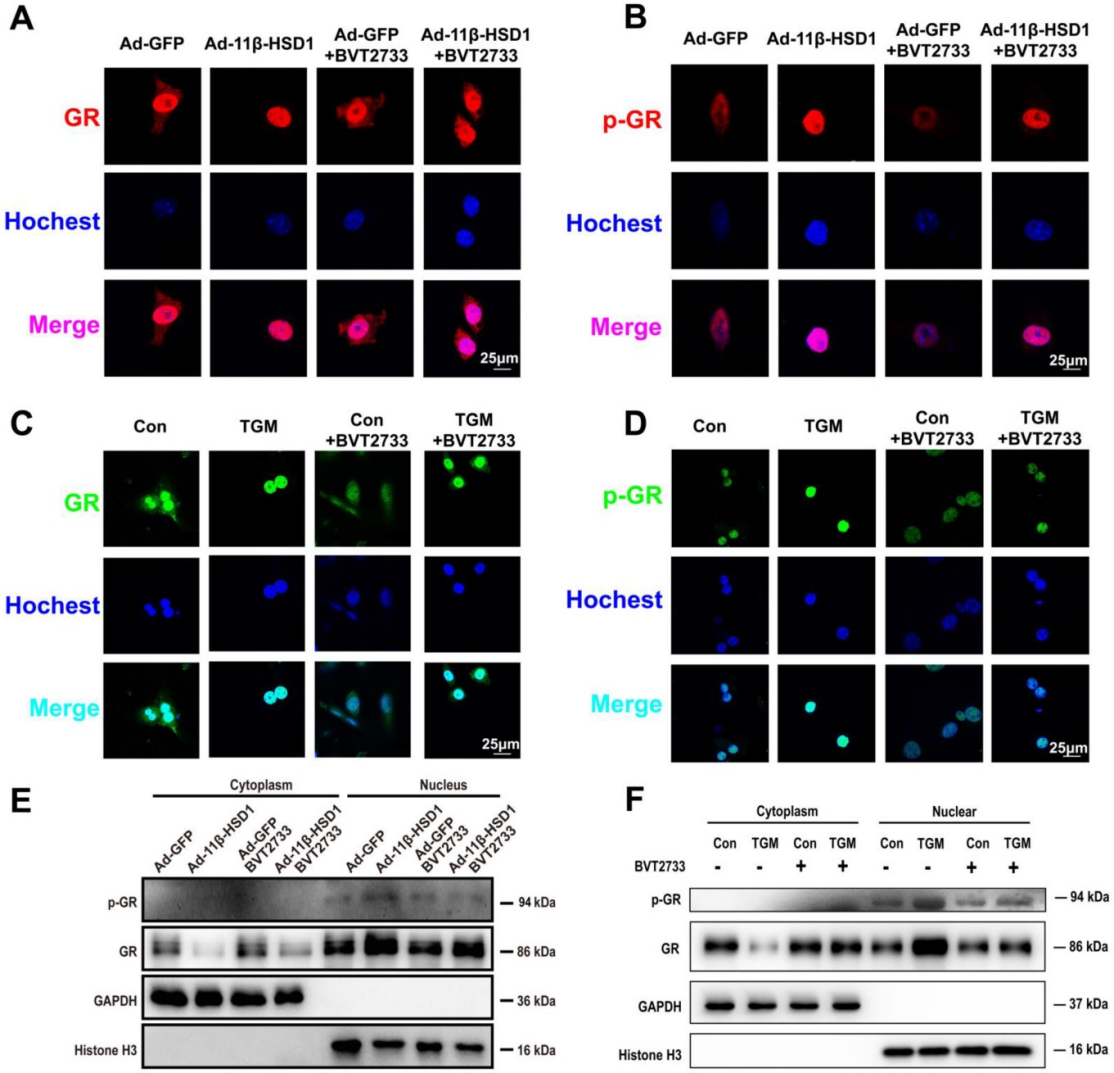
** 11β-HSD1 overexpression promoted nuclear translocation of GR and activation of p-GR.** (A) Immunofluorescence staining of the distribution of GR (red) following treatment or not with 0.25 μM BVT2733 in Hep1-6 cells overexpressing 11β-HSD1 or GFP. (B) Immunofluorescence staining of the distribution of p-GR (red) following the same manner of (A). (C) Immunofluorescence staining of the distribution of GR (green) exposed or not to 0.25 μM BVT2733 in primary hepatocytes of Con vs TGM. (D) Immunofluorescence staining of the distribution of p-GR (green) following the same manner of (C). (E) Western blot showing the levels of the GR and p-GR proteins in the cytoplasm and nucleus of Hep1-6 cells from (A). (F) Western blot showing the expression levels of the GR and p-GR proteins in cytoplasm and nuclei of primary hepatocytes from (C).

In order to determine if 11β-HSD1 activates lipogenesis in a GR-dependent manner, we performed immunofluorescence staining on Hep1-6 cells overexpressing 11β-HSD1 or GFP, as well as in primary hepatocytes of TGM or Con. We found increased GR expression and phosphorylation in the nuclei of Ad-11β-HSD1-transfected Hep1-6 cells (Figure 6A-B, respectively) and in the primary hepatocytes of TGM (Figure 6C-D. respectively), which was reversed by BVT2733 (Figure 6A-D). The nuclear/cytoplasmic separation assay further validated that GR and p-GR both accumulated in the nuclei of Hep1-6 cells overexpressing 11β-HSD1 (Figure 6E), as well as in the nuclei of primary hepatocytes from Con and TGM (Figure 6F). In general, our experiments indicated that overexpression of 11β-HSD1 led to the nuclear translocation of GR, suggesting that the effects of 11β-HSD1 on lipogenesis were associated with the activation of GR, which subsequently activated the lipid biosynthesis pathway.

### The proposed molecular mechanism by which 11β-HSD1 induces lipid accumulation through the GR/gp78/Insig2/SREBP1 pathway

**Figure 7:**
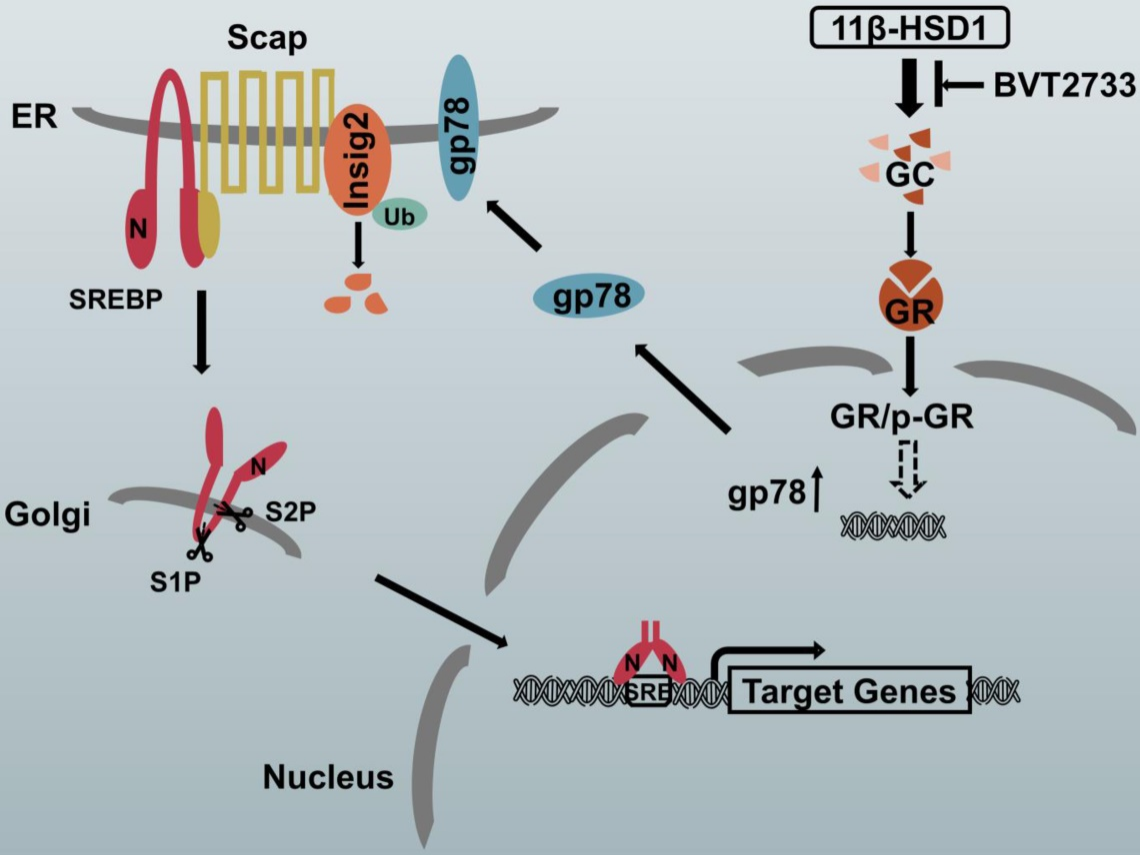
Schematic diagram of the putative mechanism by which 11β-HSD1 mediates lipid accumulation.

In hepatocytes, local GCs are activated by 11β-HSD1 overexpression, leading to the nuclear translocation of GR and an increase in p-GR, which subsequently activates the downstream lipogenesis regulatory cascade. In this study, we propose that there is a relationship between GR and gp78, in which the activation of GR up-regulates the expression of gp78, a membrane-anchored ubiquitin ligase that mediates the ubiquitination and subsequent proteasomal degradation of Insig. In our study, Insig2 was more sensitive to the gp78 response in the GC-initiated GR pathway. Under normal circumstances, SREBPs are inserted into the endoplasmic reticulum (ER) membrane as inactive precursors that can bind the SREBP cleavage-activating protein (Scap). In cells overexpressing 11β-HSD1, the SREBP-Scap complex separated more rapidly from the ER and trafficked to the Golgi complex, compared to the rate of exit from the ER observed in control mice. Once localized to the Golgi complex, Golgi-localized site-1 protease (S1P) and site-2 protease (S2P) cleave the SRBEP/Scap complex, releasing the SREBP N terminus from the membrane. The mature SREBP is then targeted to the nucleus, where it activates the transcription of genes, such as FAS, SCD1, and ACC1. The presence of BVT2733 can inhibit the activity of 11β-HSD1 and block the downstream regulatory mechanism (Figure 7).

## Discussion

A pathological increase in hepatic lipogenesis is one of the primary mechanisms underlying the development of NAFLD. Recent studies have confirmed that 11β-HSD1 is necessary for intracellular GC signalling^34^, in addition to its reported associations with lipogenesis and the development of NAFLD^35^. In our previous studies, we found that 11β-HSD1 had many effects on hepatic lipid metabolism, while inhibition of 11β-HSD1 resulted in increased energy expenditure^36^. To refine our understanding of the mechanisms by which 11β-HSD1 affects lipogenesis, in this present study we used both *in vitro* (Hep1-6 cells) and *in vivo* (hepatocyte-specific 11β-HSD1 transgenic mice) models to investigate the pathophysiological relationship between lipid accumulation in liver and 11β-HSD1 expression. Consistent with our previous work, we found that triglyceride accumulated in liver tissue and hepatocytes after overexpression of 11β-HSD1, and that the lipogenesis pathway was activated by 11β-HSD1 overexpression, specifically. We observed varying increases of SREBP1, FAS, SCD1, ACC1 and gp78, while decrease of Insig2. In addition, 11β-HSD1 promoted translocation of GR to the nucleus via activating GCs, resulting in activation of the downstream lipogenic pathway. Our current results also identified gp78 as an E3 ubiquitin ligase that initiated the GC/GR-accelerated degradation of Insig2, thus activating lipogenesis.

In NAFLD, hepatic lipid uptake and DNL are increased, whereas the compensatory increases in the oxidation of fatty acids, which does not adequately normalize lipid levels^37^, ^38^. Here, we found a slight increase in PPARα, which indicated that β-oxidation was occurring, although no obvious changes were observed in other lipid-related pathways, including lipid uptake, secretion, and hydrolysis. These results are consistent with previous research results^10^, ^36^.

Results from previous research showed that transgenic mice selectively overexpressing 11β-HSD1 in adipose tissue exhibited a full metabolic syndrome, with visceral obesity, insulin-resistant diabetes, and hypertension^39^. Another study using transgenic mice with hepatocyte-specific increases in 11β-HSD1 concluded that this was likely related to pathogenesis of fatty liver, insulin resistance and hypertensive syndromes^40^. In addition, recent studies showed that mice deficient in this enzyme were resistant to diet-induced obesity, increased insulin and leptin sensitivity^41^. In clinical and preclinical studies, 11β-HSD1 inhibitors were shown to potentially exert major pharmacological effects in metabolically active tissues^42^. Similar results were obtained using the 11β-HSD1 inhibitor BVT2733, which showed high potency in the inhibition of mouse 11β-HSD1 enzyme^43^. In our experiments, we found that BVT2733 reversed the effects of 11β-HSD1 on the lipid biosynthetic pathway gene expression, resulting in a reduction in triglyceride content and decreased expression of SREBP1, FAS, SCD1, ACC1 and gp78. Overall, our data are consistent with prior studies, thus demonstrating the impact of 11β-HSD1 on lipid accumulation in liver.

The role of SREBP-1c in the pathogenesis of fatty liver has been previously explored using different *in vivo* animal models^44^. Overexpression of SREBP1 in transgenic mouse livers led to the development of classic fatty liver disease due to increased lipogenesis^13^, ^45^. However, in contrast, inactivation of the SREBP1 gene in *ob/ob* mice resulted in a 50% reduction of liver triglyceride content^46^. Similarly, previous studies demonstrated that short-term suppression of SREBP1 expression ameliorated diet-induced NAFLD^47^. SREBP1 is an essential transcription factor in the regulation of lipid metabolism and energy storage due to the direct up-regulation of lipogenic enzymes, including ACC1, FAS and SCD1 during lipid accumulation^48^. Our results suggest that 11β-HSD1 overexpression enhanced lipid synthesis concurrently with up-regulation of the same lipogenic genes, as well as SREBP1. By contrast, 11β-HSD1 inhibition decreased lipid accumulation as well as the expression of FAS, SCD1 and ACC1, but had little effect on CPT1α and PPARα expression, both of which function as major regulators of fatty acid β-oxidation in liver^7^. Therefore, 11β-HSD1 ostensibly promotes lipid accumulation via lipogenesis but does not increase the levels of fatty acid oxidation in hepatocytes.

Rivera *et.al* proposed that GR may mediate the degradation of Insig2a^49^, and other reports have supported this hypothesis with data showing that the knockout of gp78 led to an increase in Insig1/2 levels^17^, ^19^. Considering these findings, we speculated in our study that gp78 potentially involved in the degradation of Insig1/2 via GR regulation with a certain way. We investigated differences in the expression of gp78, Hrd1^50^ and Ufd1^51^ during NAFLD (data not shown) and only found changes in the expression of gp78. Although originally identified as a receptor of autocrine motility factor (AMFR), hepatic gp78 was subsequently shown to be essential for regulating lipid and energy metabolism^52^. The gp78 enzyme was then characterized as a RING-dependent, ER membrane-anchored E3 ligase that catalyses the ubiquitination of certain folded proteins and therefore may function as a metabolic regulator by modulating the levels of proteins involved in lipid metabolism, such as HMGCR and Insig1/2^17^, ^53^, ^54^. In order to verify whether GR regulates gp78, we first performed a dual-luciferase reporter assay and found GR did not activate promotor of gp78 (data not shown). Then GR was knocked down by siRNA in primary hepatocytes and the decrease of GR would lead to the decrease of gp78 (Supplement Figure 1), which showed that GR did have a regulatory effect on gp78. The underlying molecular mechanism will be our next research direction.

Some studies have indicated that disruption of the gp78 gene in mice led to the development of non-alcoholic steatohepatitis and spontaneous hepatocellular carcinoma in aged mice^52^. However, some studies have reported contradictory results in which hepatocyte-specific gp78 knockout mice were lean and had lower blood and tissue lipid levels^19^. Furthermore, the gp78 knockout mice were resistant to normal chow diet-induced and western-type diet-induced obesity due to the suppression of SREBP via upregulation of its negative regulators, Insig1/2^19^. Possible explanations include the possibility that gp78 gene knockout in whole mice might overcome the metabolic stress observed during embryonic developmental stages and early growth in hepatocyte-specific gp78 knockout mice.

Other previous work has shown that when cells were depleted of sterols, the Scap-SREBP complex dissociated from Insig1/2, leading to its ubiquitination and degradation^18^. This dissociation allowed translocation of the Scap-SREBP complex into the Golgi complex, where SREBP was converted to its nuclear form, thus accelerating the transcriptional activation of cholesterol biosynthesis and uptake genes^18^. A reduction in Insig1/2 transcript levels has also been shown to accelerate adipocyte differentiation and to lead to enhance lipogenesis and, consequently, lipid accumulation^55^. Here, we found that the ubiquitin ligase gp78 was required for Insig2 ubiquitination-mediated degradation in Hep1-6 cells, primary hepatocytes, and transgenic mouse liver tissue with hepatocyte-specific expression of 11β-HSD1, and lipid accumulation was attenuated when knocking down gp78 in primary hepatocytes of transgenic mice, which supported that gp78 played a key role in lipid accumulation induced by 11β-HSD1 overexpression. Future study will involve more detailed characterization of gp78 activity and its specific role in lipid accumulation during NAFLD.

It is also germane to mention that hepatocyte-specific disruption of GR has been shown to improve the steatosis phenotype in fatty liver mouse models^56^. Although these results indirectly support our data, recent research has revealed that Insig2 levels increased during fasting^49^, with circulating GCs thus activating GRs to limit lipid biosynthesis. This proposed pathway was inconsistent with our data, and the results may be due to the use of differences in the experimental model systems, variations in nutrient status, or a combination of both factors. Our results demonstrated that GR was phosphorylated and translocated to the nucleus after 11β-HSD1-induced local GC activation, thereby indicating the relationship between GR/gp78/Insig2/SREBP1 in lipid metabolism.

## Supplementary Material

Supplementary figures and materials and methods.

## Conclusions

In conclusion, this study demonstrated the role of 11β-HSD1 in lipid accumulation, both *in vitro* using Hep1-6 cells and *in vivo* using a transgenic mouse model with hepatocyte-specific expression of 11β-HSD1. Overexpression of 11β-HSD1 activated GC-mediated nuclear translocation of GR, leading to the regulatory induction of lipid biosynthesis by SREBP1 through gp78-catalzyed degradation of Insig2. This process was effectively halted by the selective inhibitor BVT2733. These findings revealed the regulatory mechanisms by which 11β-HSD1 participates in lipid biosynthesis pathway and provides new potential diagnostic and therapeutic targets of metabolic diseases.

### Funding Statement

This work was supported by the CAMS Innovation Fund for Medical Sciences (CIFMS) (2021-I2M-1-008), National Key R&D Program of China (2018YFC2000100), National Natural Science Foundation of China (No. 81970739, 81471071, 81270948) and Beijing Natural Science Foundation (7212086).
