# Supplementary material for: The GR-gp78 Pathway is involved in Hepatic Lipid Accumulation Induced by Overexpression of 11β-HSD1
Source: Int J Biol Sci. 2022 Apr 30;18(8):3107–21. doi: 10.7150/ijbs.42376 (PMC9134908; doi:10.7150/ijbs.42376)
Supplement: Supplementary file 1 — Supplementary figures and materials and methods. [file ijbsv18p3107s1.pdf]

Supplemental Information

The GR-gp78 Pathway is Involved in Hepatic Lipid Accumulation Induced by Overexpression of 11β-HSD1

Mengliang Hu, Tingting Han, Qiyu Pan, Dongsheng Ni, Fengyi Gao, Liying Wang, Hangjiang Ren, Xiaoyi Zhang, Haoyun Jiao, Yuefeng Wang, Dapeng Dai, Yong Man, Weiqing Tang, Yue Sun, Wei Li* and Jian Li*, Guoping Li*

Supplement Figure 1

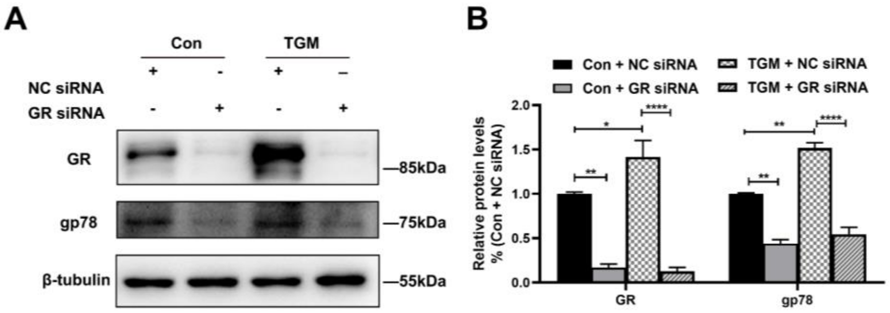

C

	sense	antisense
NC siRNA	5'-UUCUCCGAACGUGUCACGUTT-3'	5'-ACGUGACACGUUCGGAGAATT-3'
GR siRNA	5'-GCAGCAGAGGAUUCUCCUUTT-3'	5'-AAGGAGAAUCCUCUGCUGCTT-3'

Supplement Figure 1: Gp78 was decreased when knocked down of GR with siRNA in primary hepatocytes of hepatocyte-specific 11β-HSD1 transgenic mice (TGM) and the control group (Con). (A) Western blot showing the expression of GR and gp78 in primary hepatocytes of Con vs TGM after knocking down GR and (B) relative protein levels. (C) GR siRNA sequence (designed and synthesized from Genepharma (Shanghai, China)). (n = 3, *P<0.05, **P<0.01, ****P<0.0001).

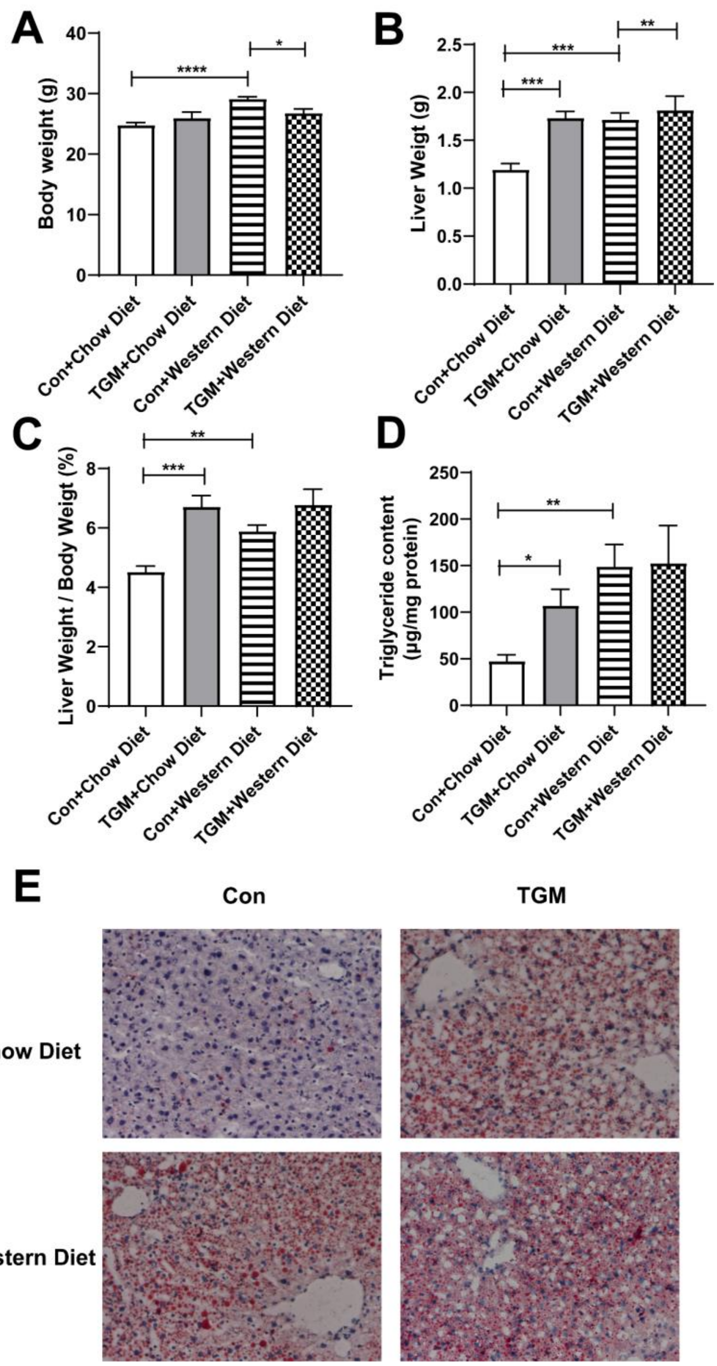

Supplement Figure 2: The phenotype of hepatocyte-specific 11β-HSD1 transgenic mice (TGM) and the control group (Con) fed with western diet. (A) Body weights (n = 5). (B) Liver weights (n = 5). (C) The statistical analysis of liver weight/body weight ratios (n = 5). (D) Measurement of triglyceride contents in livers of Con vs TGM (n = 5). (E) Oil red O staining of liver tissues. *P<0.05, **P<0.01, ***P<0.001.

Methods and Materials

siRNA transfection

The experimental procedures were conducted in the main text.

Mice fed with western diet

The western diet (H104101, fat 40% kcal, carbohydrate 43% kcal, protein 17% kcal, kcal/g = 4.7) was purchased from Hua Fukang Biological Technology Co., Ltd. Western diet feeding started at 6 weeks of mice age and continued for 12 weeks. Mice were fasted 5 ~ 6 h before sacrificed. The experimental procedures of liver triglyceride contents and Oil red O staining were conducted in the main text.
